# Mechanism of Interleukin-4 Reducing Lipid Deposit by Regulating Hormone-Sensitive Lipase

**DOI:** 10.1038/s41598-019-47908-9

**Published:** 2019-08-19

**Authors:** Ming-Yuh Shiau, Pei-Hua Chuang, Ching-Ping Yang, Chiao-Wan Hsiao, Shu-Wen Chang, Kai-Yun Chang, Tsung-Ming Liu, Huan-Wen Chen, Cheng-Chieh Chuang, Sheau-Yun Yuan, Yih-Hsin Chang

**Affiliations:** 1 Department of Nursing, College of Nursing, Hungkuang University, Taichung, Taiwan; 2 Department of Biotechnology and Laboratory Science in Medicine, National Yang-Ming University, Taipei, Taiwan; 3 Program in Molecular Medicine, National Yang-Ming University and Academia Sinica, Taipei, Taiwan; 4 Division of Urology, Department of Surgery, Taichung Veterans General Hospital, Taichung, Taiwan

**Keywords:** Mechanisms of disease, Molecular medicine

## Abstract

Accumulating evidence indicates that inflammation participates in the pathophysiological progress from insulin resistance, obesity, metabolic abnormalities, and type 2 diabetes mellitus. Our previous study reveals that interleukin-4 (IL-4) inhibits adipogenesis and promotes lipolysis to decrease lipid deposits by enhancing the activity of hormone sensitive lipase (HSL). The present study further dissects and characterizes the molecular mechanism of IL-4 in regulating HSL expression and lipolytic activity in the terminal differentiated 3T3-L1 mature adipocytes. Our results showed that IL-4 increased cAMP which then enhanced PKA activity and subsequent phosphorylation of HSL and perilipin. The phosphorylated HSL (p-HSL) translocated from cytoplasm to the surface of lipid droplets and exhibited lipolytic function. After being phosphorylated, p-perilipin also facilitated lipolysis through interacting with p-HSL. The *in vitro* findings were further verified by *in vivo* study in which IL-4 exhibited pro-lipolytic activity and enhanced HSL activity. In summary, the net outcome of IL-4 treatment is to reduce lipid storage by promoting lipolysis through enhancing HSL activity via cAMP/PKA pathway, the major route leading to lipolysis.

## Introduction

Obesity, characterized by an increased mass of fat, is the major risk factor leading to insulin resistance and type 2 diabetes mellitus (T2DM). Elevated circulatory free fatty acids (FFAs) are correlated with decreased insulin sensitivity^1^, which lead to impaired insulin secretion and the consequences of insulin-targeted organs such as insulin resistance, visceral obesity, and hepatic steatosis^2,3^.

Inflammation participates in the pathophysiological progress from insulin resistance, obesity, metabolic abnormalities, T2DM to the irreversible consequences of cardiovascular diseases^4,5^. Interleukin-6 (IL-6) and tumor necrosis factor-alpha (TNF-α) are the most extensively studied cytokines associated with metabolic disorders^6–8^. Nevertheless, the roles and involvement of other cytokines in energy homeostasis and metabolism are much less elucidated.

The pleiotropic cytokine interleukin 4 (IL-4) harbors diverse effects on a variety of target cells^9–11^. IL-4 exerts anti-inflammatory function by reducing the production and activities of important pro-inflammatory cytokines. In addition to almost completely inhibiting the production and secretion of IL-1β and TNF-α^12^, IL-4 suppresses IL-6 secretion and activities^13^. We therefore started to study the roles and regulation of IL-4 in energy homeostasis, aiming to explore its potential activities in modulating metabolism.

In this context, significant associations between *IL-4* and *IL-4R* with high density lipoprotein-cholesterol (HDL-C) and T2DM are identified^14,15^. IL-4 improves *in vivo* insulin sensitivity and glucose tolerance while inhibits lipid accumulation in fat tissues, leading to decreased weight gain and fat mass^16^. In addition, IL-4 is suggested to indirectly mediate energy homeostasis through regulating adipose tissue-derived adipokines^16^. These results reveal the novel functions of IL-4 in glucose/lipid metabolism by improving insulin sensitivity, glucose tolerance, and inhibiting lipid deposits, as well as deviating adipocyte behaviors to catabolism^17^. We further identify that IL-4 inhibits adipogenesis and promotes lipolysis to decrease lipid accumulation in adipocytes by enhancing the activity and translocation of hormone sensitive lipase (HSL)^18^. Collectively, IL-4 harbors pro-lipolytic capacity by inhibiting adipocyte differentiation and lipid accumulation, as well as promoting lipolysis in mature adipocytes to decrease lipid deposits.

The above findings not only disclose the metabolism-regulating functions of IL-4, but also elucidate the interactions among cytokines/immune responses, insulin sensitivity and lipid metabolism. Additionally, these observations support our speculation that IL-4 harbors the activity to attenuate the development of insulin resistance and the subsequent progression to metabolic abnormalities. The present study aimed at examining the molecular mechanism of IL-4 regulating HSL activity for further addressing the involvement of IL-4 in lipid metabolism.

## Methods

### Materials

Antibodies against HSL (#4107), phosphorylated-HSL (p-HSL) at Ser563 (p-HSL Ser563, #4139), p-HSL Ser565 (#4137), p-HSL Ser660 (#4126), perilipin (#3470), phospho-(Ser/Thr) protein kinase A (PKA) substrates (p-PKA substrates, #9621), and β-actin (A5441) were purchased form Sigma (St. Louis, MO, USA); AMP-activated protein kinase (AMPK; 07-350SP) from Calbiochem (Merck Millipore, Billerica, MA, USA); p-AMPKThr172 (E11-0003A) from EnoGene Biotech; secondary antibodies: goat anti-mouse IgG HRP conjugate (71045) from Merck Millipore (Billerica, MA, USA) and EasyBlot anti rabbit IgG (GTX221666-01) from GeneTex. Trizol Reagent was purchased from Life Technology (Invitrogen 15596-018; Carlsbad, CA, USA); ECL reagent from Calbiochem (WBKLS0500); 3-isobutyl-methylxanthine (IBMX; I5879), dexamethasone (Dex; D1756), and insulin (I5523) from Sigma; HSL siRNA from BioTools, Inc. Chow diet (LabDiet 5010; with 13 kcal% fat) and high fat diet (HFD, D12492; Rodent Diet with 60 kcal% fat) were purchased from Research Diet Inc. (NJ 08901 USA).

### 3T3-L1 cell culture and treatment

3T3-L1 pre-adipocytes were allowed to differentiate into mature adipocytes as described^17,18^. In brief, the preadipocytes were cultured in DMEM containing 10% calf serum (Hyclone Laboratories, South Logan, Utah, USA). 2-day postconfluent (designated day 0), cells were induced to differentiate by adding 0.5 mM IBMX, 1 μM Dex, and 10 μg/ml insulin in 10% FBS for 2 days. The cells were then cultured in DMEM supplemented with 10% FBS and 5 μg/ml insulin for the next 6 days to allow the cells become terminally differentiated mature adipocytes^17,18^. For IL-4 treatment, *Hsl* mRNA levels were examined after the mature adipocytes were exposed to 10 ng/mL IL-4 for different time intervals after serum starvation for 2 h. For H89 experiments, mature adipocytes were pre-incubated with 20 μM H89 for 1 h and then treated with IL-4. To assess protein stability, mature adipocytes were treated with 10 μg/mL cycloheximide (CHX; C7698 from Sigma) in the presence or absence of IL-4. At the given time points, cells lysates were harvested and analyzed by Western blotting. Protein degradation rates were quantified by densitometry using time point zero as 100%.

### Western blot analysis

Cell lysates were prepared in RIPA buffer containing protease inhibitors as described^16,18,19^. In brief, total cell lysates were extracted at 4 °C by lysis buffer containing proteinase and phosphatase inhibitors. For animal study, protein extracts from epididymal fat and liver were obtained respectively after the tissues were homogenized using T-PER tissue protein extraction reagent (8510; Pierce, Rockford, IL, USA) supplied with phosphatase (04906837001) and protease inhibitors (04693116001; Roche, Indianapolis, IN, USA). Extracts were centrifuged at 14,000 rpm at 4 °C for 15 min and supernatants were collected. Sixty μg of cell extracts were subjected to SDS-PAGE, transferred to PVDF membrane and blotted with specific primary antibodies. For detection, membranes were incubated with secondary antibodies (1:10,000) for 1 h, followed by being visualized with ECL regent and exposed to X-films. The blot was quantified by Multi Gauge Version 3.0.

### Real-time quantitative PCR

About 2 μg of total RNA isolated by TRIzol reagent was reversed transcribed with Improm-II Reverse Transcription Kit (A3800; Promega, Madison, WI, USA). Real-time PCR was performed using the Applied Biosystems SYBR Green Realtime PCR Master Mix and StepOnePlus™ Real-Time PCR System using the following primers: *Hsl* (XM_021167213; 5′-CCGCTGACTTCCTGCAAGAG-3′ and 5′-CTGGGTCTATGGCGAATCGG-3′; annealing temperature 60 °C, PCR product size 213 bp,), and 18 s *rRna* (NR_003278; 5′-CGGCTACCACATCCAAGGAA-3′ and 5′-GCTGGAATTACCGCGGCT-3′; annealing temperature 60 °C, PCR product size 187 bp).

### Hsl promoter assay

A ~1.5 kb *Hsl* promoter region was amplified and subcloned into pGL4 vector (E6651; Promega, Wisconsin, USA) to generate *Hsl* promoter-carrying pGL4 vector (*Hsl*-p-pGL4). Cells with 80% confluence were transfected with 1 μg of *Hsl*-p-pGL4 using PolyJet^TM^ reagent (SL100688; SignaGen), and co-transfected with 1 ng of pRL containing Renilla luciferase-encoding gene with Lipofectamine 2000 (11668027; Lifetechnologies, Massachusetts, USA) for normalization. The promoter activity was analyzed using Dual-Luciferase® Reporter 1000 Assay Systems (E1980; Promega). Light counts from luciferase and renilla were obtained in Vitor^2^ bioluminescence counter (PerkinElmer).

### Glycerol and cAMP measurement

Cells were incubated in serum free DMEM with or without IL-4 and/or isoproterenol (Iso, 1351005; Sigma) treatment, then glycerol contents in the culture medium were measured. For analyzing cAMP, cells were pre-incubated with 0.5 mM IBMX for 0.5 h to block phosphodiesterase activity for preventing the produced cAMP from being hydrolyzed, then stimulated with 10 μM Iso and IL-4 for another 1 h. Concentrations of glycerol in the culture medium and intracellular cAMP were analyzed, respectively, by Free Glycerol Determination Kit (F6428; Sigma) and Screen Quest™ Colorimetric ELISA cAMP Assay Kit (20249; BMassay).

### Hsl small interfering RNA (siRNA)

Cells were transfected with 25 nM of either *Hsl*-specific (HSL-mus-276; 5′-GCCAACGGAUACCGUAGUUTT-3′ and 5′-AACUACGGUAUCCGUUGGCTT-3′) or non-specific scramble siRNA (5′-UUCUCCGAACGUGUCACGUTT-3′ and 5′-ACGUGACACGUUCGGAGAATT-3′) on day -2 by TransIT-X2 Dynamic Deliver System (MIR6000; Mirus Bio LLC), then induced to differentiate on day 0 by supplementing another dose of siRNA and incubated for additional 48 hr. HSL was analyzed by Western blotting to evaluate the knockdown efficiency.

### Animal experiments

Animal experiments were conducted as described^16^. Male C57BL/6 mice were first randomly grouped and fed with Chow or HFD to induce insulin resistance. After 8 wk, Chow- and HFD- feeding mice were further randomly grouped to receive *i.p*. administration with either PBS or recombinant IL-4 (1,000 pg per mouse, 550067; BD Pharmingen) every other day for 8 weeks. Protein extracts from the harvested primary epididymal fat and liver tissues were obtained as described. Serum glycerol and FFAs were measured after overnight fast using Free Glycerol Determination Kit and Free Fatty Acid Quantification Colorimetric/Fluorometric Kit (K612; BioVision, Mountain View, CA, USA), respectively. Animal protocols were reviewed and approved by the Institutional Animal Care and Use Committee, National Yang-Ming University (IACUC approval no. 1001244), with all methods performed in accordance with the relevant guidelines and regulations.

### Statistical analysis

Each experiment was carried out at least three times. Data were presented as mean ± S.E.M. Significant difference between groups was analyzed by two-tailed unpaired Student *t*-test or one-way ANOVA followed by Tukey’s honest significant difference post hoc tests when more than 2 groups of data sets were assessed. Statistical difference was defined as *p* < 0.05 for all tests.

## Results

### Effects of IL-4 on HSL expression, stability and degradation

**Figure 1:**
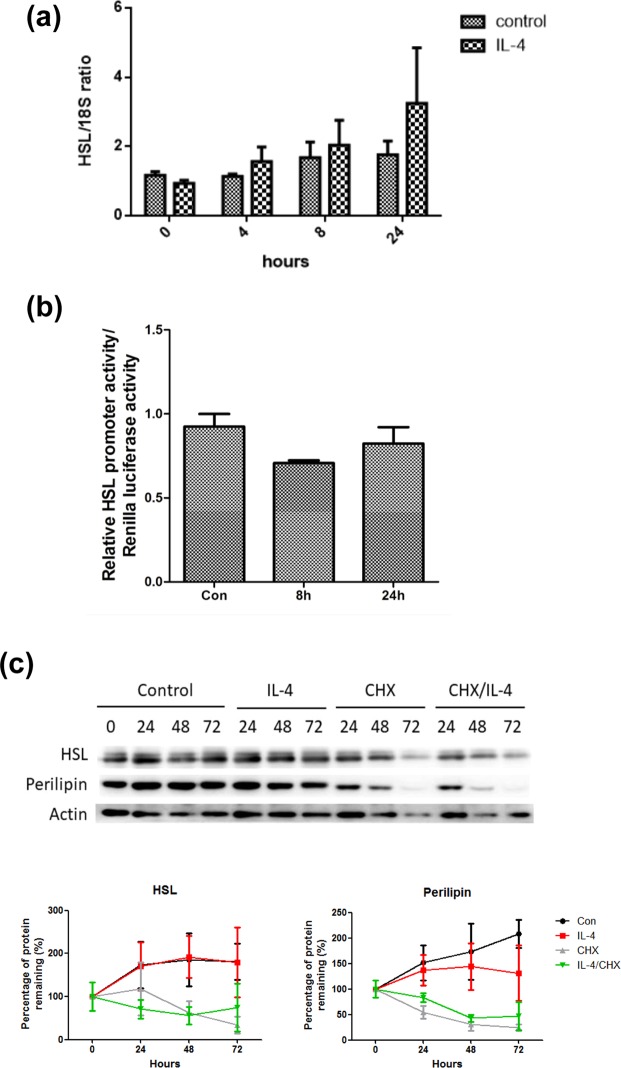
Effects of IL-4 on HSL expression, stability and degradation (**a**) 3T3-L1 mature adipocytes were serum-free starved for 2 hr then treated with IL-4 (10 ng/mL) for the time indicated, then *Hsl* mRNA was analyzed by real-time PCR. Gene expression was normalized to 18 s rRNA (n = 7). (**b**) *Hsl* promoter activity under IL-4 treatment was analyzed by dual-luciferase reporter assay as detailed described in Methods. (**c**) Mature adipocytes were treated with IL-4 (10 ng/mL) or CHX (10 μg/mL) for the indicated time after 2 hr of serum starvation. The half-lives of HSL and perilipin were analyzed by Western blotting (n = 11). The data were shown by grouping of blots cropped from different parts of the same gel.

A trend of up-regulating *Hsl* mRNA by IL-4 in a time-dependent manner was noticed, however, the results did not reach statistical significance **(**Fig. 1a**)**. Nevertheless, *Hsl* mRNA in cells with IL-4 treatment was increased about 2 folds after 24 hr. We then tested if the increased *Hsl* mRNA was resulted from the elevated transcriptional activity of *Hsl* promoter. *Hsl* promoter activities remained consistent under IL-4 treatment **(**Fig. 1b**)**. The data suggest that IL-4 tends to facilitate *Hsl* mRNA expression although no significant difference was identified.

We next probed if IL-4 modulated HSL protein expression and stability. HSL was examined after the mature adipocytes were treated with IL-4 and/or CHX for the time indicated; meanwhile, lipid droplet (LD)-associated protein perilipin was analyzed as well (Fig. 1c). Neither HSL expression and stability nor its ubiquitination and lysosomal degradation (data not shown) were altered by IL-4. Although perilipin expression and half-life tended to be enhanced and prolonged respectively by IL-4, the results did not show significant difference.

### Regulation of IL-4 to HSL phosphorylation and activity

HSL activity is controlled by the dynamic balance among its phosphorylation at Ser563, Ser565 and Ser660^20^. Phosphorylation at HSL Ser563 (p-HSL Ser563) is the pre-requisite for Ser660 phosphorylation. HSL translocates to LDs surface to exert its lipolytic activity when Ser563 is phosphorylated, and acquires maximal activity when Ser563 and Ser660 are both phosphorylated^20,21^. On the contrary, the AMPK-triggered p-HSL Ser565 suppresses HSL activity^21^.

**Figure 2:**
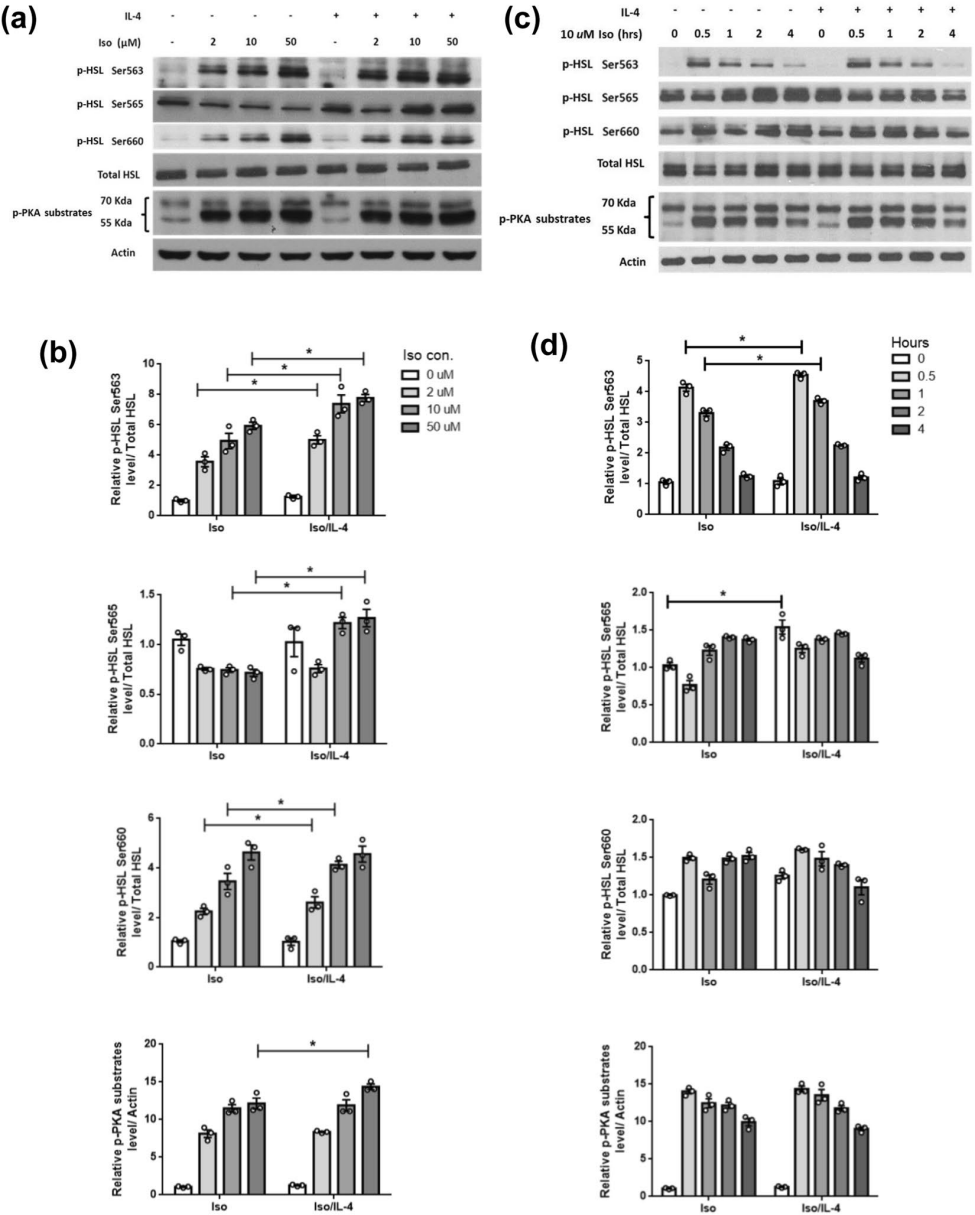
Dose and temporal effects of isoproterenol on IL-4-mediated phosphorylation of HSL, perilipin and PKA. (**a**) After 2 hr of serum starvation, adipocytes were pre-treated with 10 ng/mL IL-4 for 1 hr, followed by adding various concentrations of Iso for additional 1 hr. Then the phosphorylated protein levels were examined by Western blot. (**b**) Quantification of (**a**). (**c**) After 2 hr of serum starvation, adipocytes were treated with 10 μM Iso in the presence or absence of 10 ng/mL IL-4 for the time indicated. (**d**) Quantification of (**c**). *p < 0.05. The data were shown by grouping of blots cropped from different parts of the same gel.

To explore the molecular mechanism of IL-4 regulating HSL, p-HSL under IL-4 exposure were investigated. The β-adrenergic receptor agonist isoproterenol (Iso) dramatically increased p-HSL at Ser563/Ser660 by a dose-dependent manner and inhibited p-HSL Ser565 (Fig. 2a,b). IL-4 further boosted Iso-induced p-HSL at Ser 563/Ser660, however, the inhibitory effect of Iso on p-HSL Ser565 was attenuated by IL-4. IL-4 also significantly augmented Iso-induced phosphorylated-PKA (p-PKA) substrates.

Temporal alternations of p-HSL levels under Iso exposure were examined as well. Phosphorylated-HSL at Ser563/Ser660 reached the maximal levels after 30 min of Iso treatment, and gradually decreased thereafter (Fig. 2c,d). Amounts of p-HSL Ser563 returned to the basal level after 4 hr of treatment while p-HSL Ser660 remained high. Alterations of p-HSL at Ser563/Ser660 in the presence of combined IL-4/Iso treatment were similar to the pattern with Iso exposure.

Therefore, isoproterenol induces p-HSL at Ser563/Ser660 by a time- and dose- dependent pattern to enhance HSL activity. The net effect of IL-4 on boosting HSL activity is primarily through up-regulating p-HSL at Ser563/Ser660.

### IL-4 promotes HSL lipolytic activity through PKA pathway

**Figure 3:**
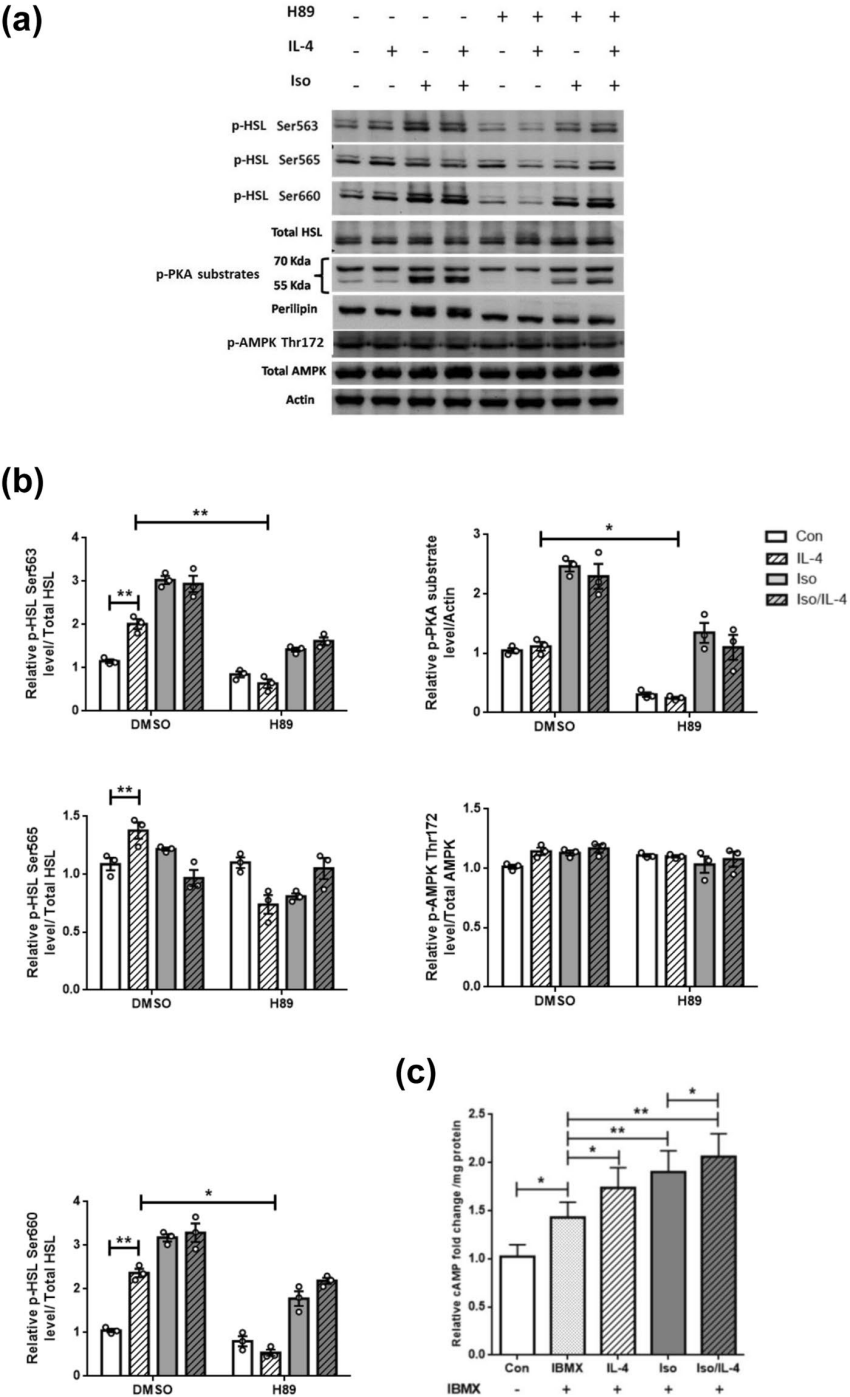
IL-4 promotes HSL activity through PKA pathway. Cells were pre-incubated in H89-contatining serum free DMEM, followed by adding IL-4 and/or Iso. (**a**) Proteins levels were analyzed by Western blotting. The data were shown by grouping of blots cropped from different parts of the same gel. (**b**) Quantification of (**a**). (**c**) Cells were pre-incubated with 0.5 mM IBMX for 0.5 h to block phosphodiesterase activity and then stimulated with 10 μM Iso and IL-4 for another 1 hr. The cell lysates were extracted for cAMP assay. *p < 0.05, **p < 0.005.

Levels of the p-HSL under Iso and/or IL-4 treatment were analyzed in the presence of PKA activity inhibitor H89. H89 not only significantly inhibited the elevated p-HSL at Ser563/Ser660 but also decreased p-PKA substrates induced by Iso and/or IL-4 treatment (Fig. 3a,b). The results suggest that IL-4 up-regulates HSL activity through PKA pathway. H89 also attenuated p-perilipin under Iso and/or IL-4 stimulation.

Cyclic AMP serves as the signaling molecule which determines PKA activity and AMPK status^20^. Accordingly, AMPK activity and the PKA upstream regulator intracellular cAMP contents were examined. IL-4 significantly increased cAMP contents, and Iso administration further enhanced the IL-4-induced cAMP levels (Fig. 3c). p-AMPK Thr172 remained unchanged under IL-4 and/or Iso exposure, regardless of H89 treatment (Fig. 3a,b). The data indicate that major outcome of IL-4-induced cAMP is to promote lipolysis by enhancing HSL activity through PKA pathway.

### IL-4 promotes lipolysis

**Figure 4:**
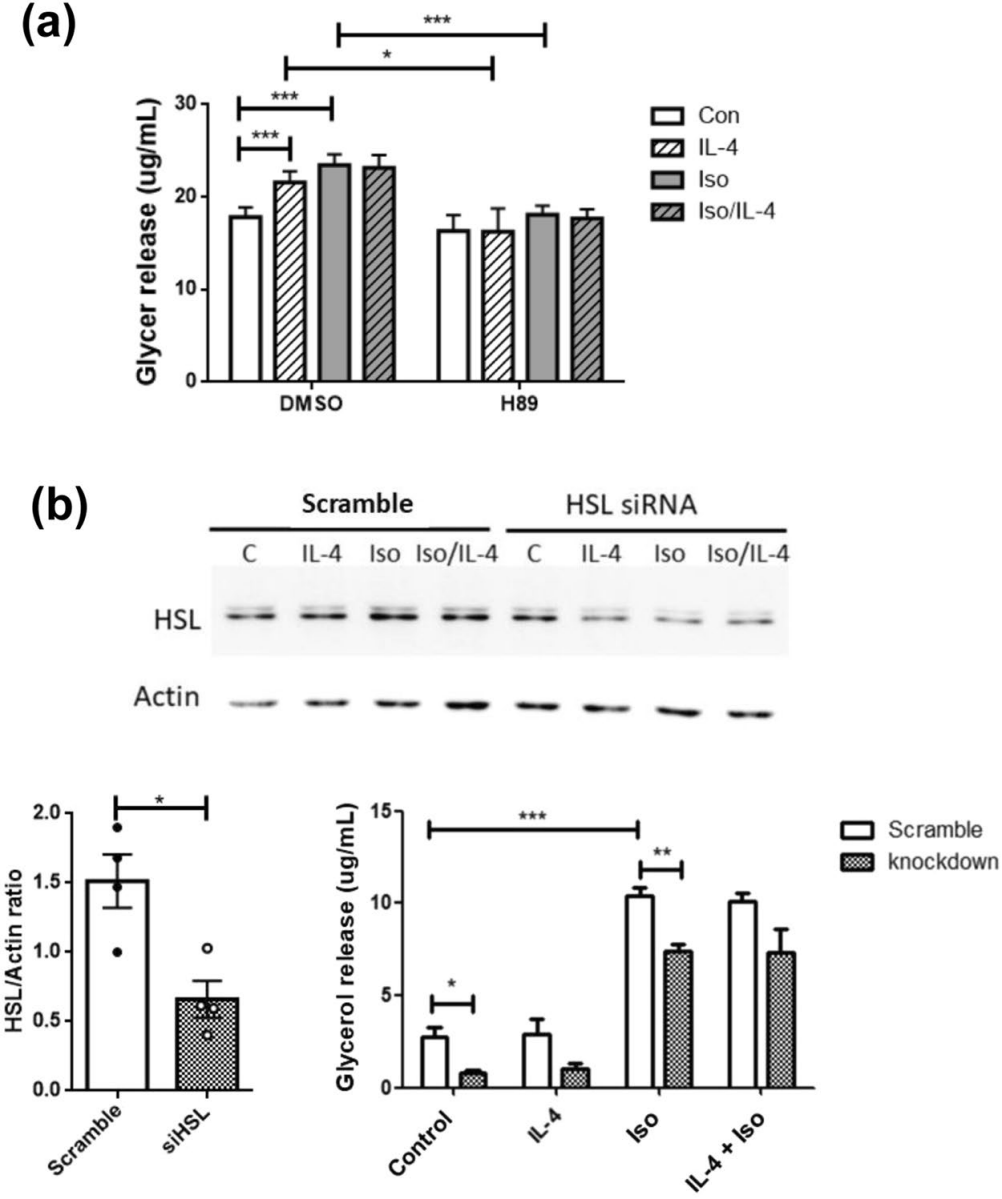
IL-4 promotes lipolysis. (**a**) Cells were pre-incubated in H89-contatining serum free DMEM, followed by adding IL-4 and/or Iso. *p < 0.05, **p < 0.005, ***p < 0.0005. (**b**) Lipolysis-promoting activity of IL-4 was attenuated by HSL knockdown (n = 4). Contents of glycerol released into cell media were measured as described in Methods. *p < 0.05, **p < 0.01, ***p < 0.001. The data were shown by grouping of blots crop*p*ed from different parts of the same gel.

Triacylglycerols stored in adipocytes are processed to release FFAs and glycerol after sequential lipolytic events in response to energy needs. Therefore, glycerol released by adipocytes under IL-4 and/or Iso treatment was quantified. Glycerol was significantly increased in the presence of IL-4 treatment (Fig. 4a). The increased glycerol release under IL-4 and/or Iso exposure supports that IL-4 promotes lipolysis by mediating HSL activity, which was further verified by the attenuation of increased glycerol release by H89. Furthermore, the synergistic effect of IL-4 to promote Iso-induced lipolysis was abolished by HSL-specific siRNA (Fig. 4b). These data strongly support that IL-4 enables HSL to exert the lipolytic activity by promoting p-HSL Ser563/Ser660 through PKA pathway.

### Regulation of IL-4 to HSL activity and lipolysis *in vivo*

**Figure 5:**
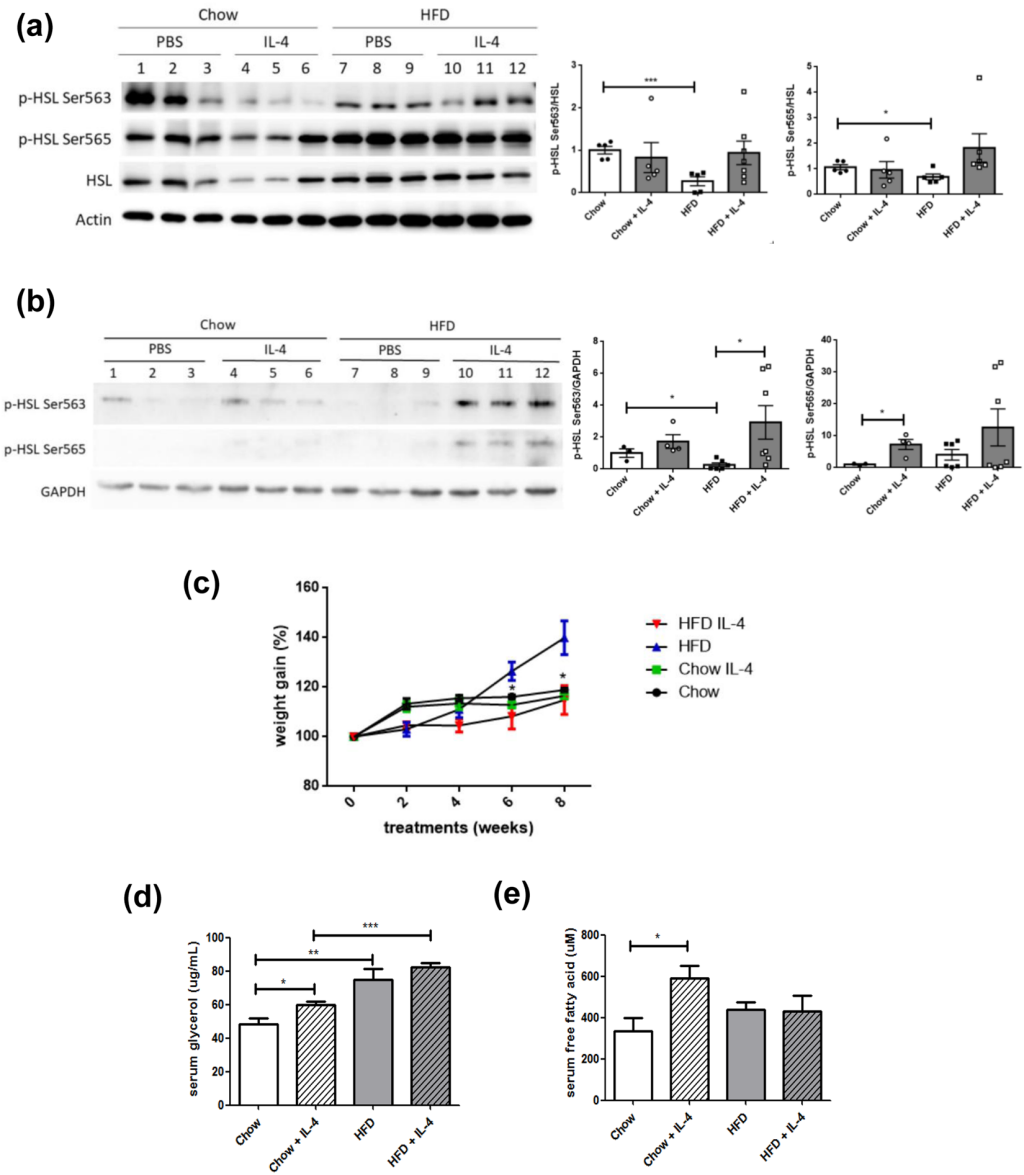
Regulation of lipolysis and hepatic HSL activity by IL-4 *in vivo*. Representative results showing the alterations of p-HSL in adipose tissue (**a**) and liver lysates (**b**) obtained from mice receiving IL-4 injection with either standard chow (Chow) or high fat diet (HFD) as described in Methods. The data were shown by grouping of blots cropped from different parts of the same gel. (**c**) Results of the temporal alterations in body weight gain of mice receiving either IL-4 or PBS fed with the HFD or chow diet. *p < 0.05, HFD v.s HFD + IL-4 mice. (**d**) Glycerol and (**e**) FFAs levels in fasting serum. n = 5, 5, 6, 7 for Chow, Chow + IL-4, HFD and HFD + IL-4 mice, respectively. *p < 0.05, **p < 0.01, ***p < 0.001.

The above *in vitro* experiments indicate IL-4 up-regulates the molecular machinery to promote HSL activity in adipocytes. We further examined the *in vivo* modulation of IL-4 to HSL for verifying the findings. Levels of p-HSL in adipose tissue and liver lysates from mice receiving *i.p*. IL-4 administration with either standard chow (Chow) or high fat diet (HFD) were analyzed. Interestingly, adipose p-HSL at Ser563/Ser565 (Fig. 5a) was significantly reduced in HFD mice, compared with their Chow counterparts. It indicates that adipose HSL activity is modulated by nutritional status. IL-4 tended to increase adipose p-HSL Ser563/Ser565, but without significant difference. While hepatic p-HSL Ser563 was significantly decreased in HFD mice compared to the Chow counterparts, no prominent changes of p-HSL Ser565 was identified (Fig. 5b). Notably, hepatic p-HSL Ser563 was significantly increased in HFD + IL-4 mice, suggesting that IL-4 enhances hepatic HSL activity via promoting p-HSL.

The alterations of mice weight gain during the entire study period were recorded. In support of our previous report^16,19^, HFD mice showed a significant lower weight gain after receiving 6 weeks of IL-4 administration (Fig. 5c). Fasting circulatory glycerol and FFAs were measured to investigate the *in vivo* lipolysis-promoting activity of IL-4. Chow + IL-4 mice had higher glycerol and FFAs (Fig. 5d,e). Whereas, IL-4 did not cause prominent changes of glycerol and FFAs in HFD mice. The observations explain the molecular events of our previous finding^16^ that IL-4-treated mice have smaller adipose mass with less lipid deposits. Besides, the data support the conclusions of our previous reports^16,19,21^ that only under physiological insulin-sensitive condition, can IL-4 fully exert its metabolic-regulating activity. Accordingly, we suggest although IL-4 exhibits pro-lipolytic activity under normal physiological condition, IL-4 alone is insufficient to improve energy homeostasis once insulin resistance had been developed.

## Discussion

**Figure 6:**
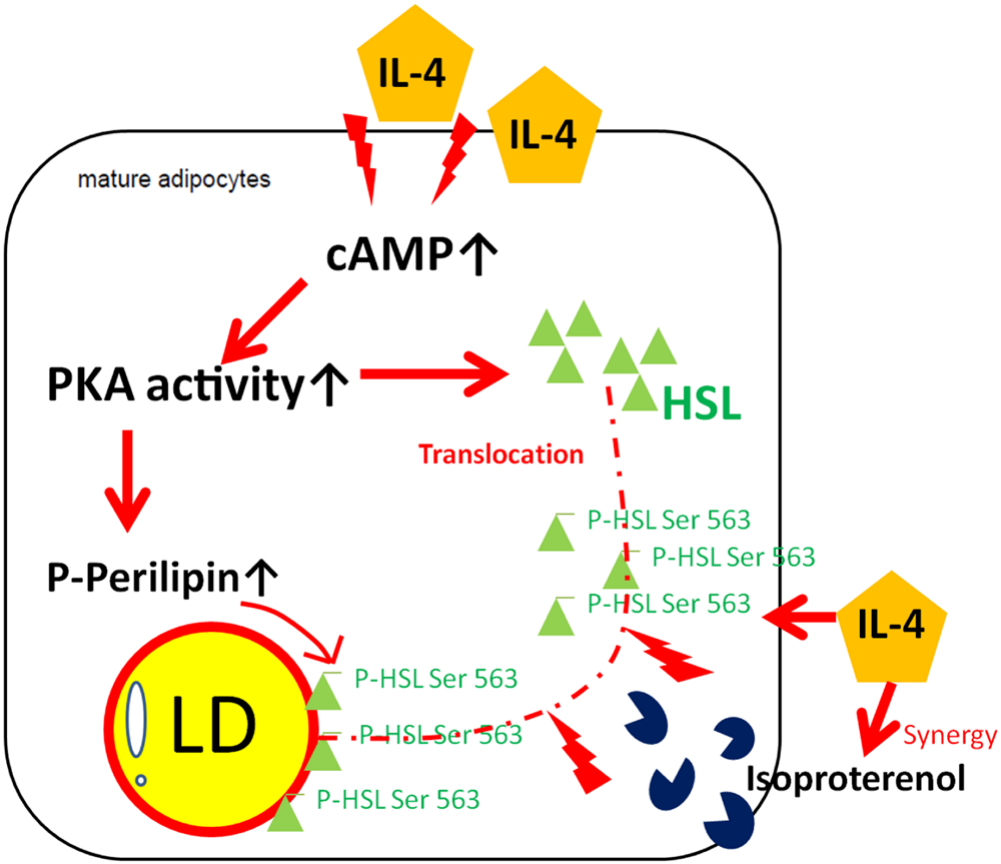
Molecular model of IL-4 regulating HSL in adipocytes. IL-4 increases cAMP in adipocytes, then the increased cAMP enhances PKA activity and subsequent phosphorylation of HSL and perilipin. HSL phosphorylated by PKA starts to translocate from cytoplasm to the surface of lipid droplets and degrades triacylglycerol. IL-4 boosts HSL phosphorylation/translocation in the presence of isoproterenol stimulation. After being phosphorylated, perilipin also promotes lipolysis through the interaction with p-HSL. In conclusion, IL-4 promotes lipolysis by enhancing HSL activity through cAMP/PKA pathway, the major route leading to lipolysis.

The molecular cascade of IL-4 regulating HSL is depicted in Fig. 6 by combining the findings from our previous report^18^ and the current study. IL-4 increases cAMP which then enhances PKA activity and subsequent phosphorylation of HSL at Ser563/Ser660, enabling HSL to exert the maximal activity. The p-HSL translocates from cytoplasm to LD surface and exhibits lipolytic function. IL-4 also facilitates HSL translocation/activity by augmenting perilipin phosphorylation. The outcome of IL-4 signal is to reduce adipocyte lipid storage by enhancing lipolysis.

HSL activity is subtly orchestrated by its phosphorylated status^22^. Our results suggest that p-HSL Ser563 is the pivotal site for IL-4 regulating HSL activity. Nevertheless, IL-4 also up-regulates p-HSL Ser565 at higher isoproterenol concentrations (Fig. 2a). We speculate that IL-4 may mediate HSL activity via unidentified signaling pathway as H89 only partially reduces p-HSL Ser565 (Fig. 3b). The other possibility is that this p-HSL Ser565-regulatory effect at higher isoproterenol doses may serve as a negative feedback regulation to counteract vigorous lipolysis for maintaining metabolic homeostasis. Nevertheless, this speculation and how IL-4 transverses its signaling to modulate p-HSL Ser565 need further investigation.

Results regarding p-HSL upon IL-4 stimulation in Figs 2 and 3 seemed inconsistent. The different adipocyte differentiation rates which result in differential lipid contents and perilipin levels among cell batches are likely the underlying reasons. Nevertheless, the IL-4-induced p-HSL Ser563 is reproducibly increased 1.5~2 folds as demonstrated in the *in vitro* results (Figs 2–4**)**, and the IL-4-enhanced HSL activity is further verified by *in vivo* experiments (Fig. 5).

In addition to adipocytes, adipose tissue also contains adipose-infiltrated macrophages (AIMs) and stromal vascular cells. AIM percentage in adipose tissue ranges from under 10% in lean mice to over 50% in extremely obese mice^23^. These AIMs also have HSL expression, however, with extremely low levels and lipolytic activity which corresponds to ~2.5% of adipocytes^24,25^. The adipose AIMs ratio was increased to approximately 20% in HFD mice with IL-4 administration (our unpublished results). Taking the consideration of the AIMs ratio and the extremely low macrophage HSL activity together, we suggest that AIM HSL expression and activity are unlikely to contribute much to the *in vivo* study results.

Inhibiting adipocyte lipolysis is one of the AMPK major effects in response to cellular energy request^26^. Intracellular cAMP either promotes lipolysis through PKA or suppresses lipolysis via activating AMPK after the cAMP is catalytically conversed to 5′AMP by phosphodiesterase. The data regarding AMPK expression and activity were not affected by IL-4 (Figs 2 and 3) reveal that IL-4-induced cAMP aims at promoting lipolysis, rather than being involved in AMPK-mediated events.

Manipulating gene expression in adipocytes has long been recognized as a challenging task. To transfect the *Hsl* siRNA into pre-adipocytes before differentiation turned out to be optimal but compromised strategy. The influences of reduced HSL to adipocyte lipolysis were successfully characterized despite certain amount of cells were died after transfection, which was reflected by the apparently decreased cell numbers/lipid contents of the mature adipocytes and thus the reduced amount of glycerol released by the transfectants (1~11 ug/mL, Fig. 4b; compared to the corresponding parental cells, 18~25 ug/mL, Fig. 4a).

The present study is not only in support of our previous findings that IL-4 harbors pro-lipolytic capacity including decreasing fat mass, suppressing adipocyte differentiation, promoting lipolysis and up-regulating the machinery accelerating ATP synthesis in mature adipocytes^16–18^, but also elucidates the underlying molecular mechanism. It strongly suggests that IL-4 improves energy metabolic efficiency by generating a favorable condition for lipolysis. This conclusion echoes the finding that mice with STAT6 deficiency have significantly less weight gain and smaller adipose deposits^27^. Furthermore, our findings provide the molecular evidence to explain that IL-4 transgenic mice contain less and smaller sized dermal fat tissues^28^, and IL-4 secretion is promoted in fatless A-ZIP/F1 diabetic mice^29^.

Splenic lymphocytes-derived IL-4 is increased in diet-induced obese mice^30^, and serum IL-4 is reduced in Sprague-Dawley rats after receiving visceral fat removal surgery^31^. Moreover, adipocyte-derived IL-4 is suggested to participate in the crosstalk between adipocytes and AIMs to improve lipid metabolism and insulin sensitivity^32,33^. Except for directly participating in the regulation of lipid metabolism, microenvironmental IL-4 may antagonize chronic inflammation in adipose tissues once insulin resistance is occurred. The locally-secreted IL-4 may be induced as a compensatory effect in metabolic imbalance for counteracting the deleterious consequences resulted from the elevated pro-inflammatory cytokines. Nevertheless, a recent study concluded that IL-4 is a lipogenesis-stimulating and lipolysis-inhibiting pro-inflammatory factor^34^. The discrepancies are very likely resulted from different experimental designing and strategies. Study from Szczepankiewicz *et al*.^34^ analyzed lipolysis and the expression of several targeted genes in primary rat mature adipocytes after the cells were *in vitro* stimulated with IL-4. They showed that IL-4 up-regulated leptin and resistin while decreased the amount of secreted adiponectin. In addition, p-HSL was down-regulated by IL-4. Accordingly, their conclusions were derived from the observations from primary adipocytes with *in vitro* IL-4 stimulation. Whereas, we explored the regulatory activity of IL-4 to energy metabolism by both *in vitro* and *in vivo* strategies. In particular, our *in vivo* animal study results are in support of the *in vitro* data^16–18^, which demonstrate that IL-4 promotes catabolism by promoting energy metabolism and lipolysis, as well as inhibiting lipogenesis.

Despite the positive protective roles of IL-4 in lipid metabolism, FFAs produced by IL-4-triggered catabolic events may be released into periphery to modulate systemic energy metabolism. Our recent study^19^ reveal that IL-4 increases hepatic triglycerides through facilitating FFAs uptake and expression/activity of lipogenic enzymes, which supports the finding that long-term uncontrolled excess FFAs in circulation are likely to result in hepatic steatosis^35^. Therefore, IL-4 may have dual roles in energy metabolism: on one hand, exerting protective function to inhibit lipid accumulation and promote catabolic efficiency; while on the other, leading to hepatic steatosis by promoting FFAs release from adipocytes into circulation. However, IL-4 may also have protective effects on hepatocytes by promoting HSL activity (Fig. 5b). Collectively, the development and progression of metabolic abnormalities reflect the outcome of the delicate and integrative dynamic effects weaved by complicated interplay/network among various cytokines, metabolic organs and intrinsic physiological factor(s).

Differentiated 3T3-L1 cells may present phenotypic features of multiple adipocyte lineages^36^. While 3T3-L1 adipocytes display gene expression profiles and basal bioenergetics of white adipocytes, the brown adipocyte characteristic UCP1-dependent uncoupled respiration would be increased upon acute norepinephrine stimulation. As a matter of fact, the possibility of IL-4 to induce browning effect of 3T3 cells was investigated in our study. The data showed that IL-4 did not participate in the regulation of adipocyte browning marker genes (unpublished observations). Accordingly, the concern whether IL-4 would trigger 3T3-L1 white adipocytes browning and therefore affect the findings and derived conclusions should be able to be excluded.

In brief, our study characterizes the molecular mechanism of IL-4 regulating lipid metabolism through mediating HSL activity. The data add novel information to the regulation of lipid energy stores and address the molecular events in the maintenance of energy homeostasis as well as the pathophysiology of metabolic abnormalities. At clinical setting, significant association between IL-4 genotypes and T2DM^14^ as well as between the genotypes of *IL-4* and *IL-4R* with HDL-C are disclosed^15^. At the animal study level, IL-4 promotes insulin sensitivity, glucose tolerance and inhibits lipid deposits^16^. At the cellular and molecular level, IL-4 enhances lipolysis by facilitating perilipin phosphorylation, promoting HSL activity/translocation and inhibiting adipocytes differentiation^18^. The present study further elucidates the underlying mechanisms. By combining the findings from our serial reports, the contour of IL-4 in regulating energy metabolism, and therefore, the process from getting obesity, insulin resistance and diabetic onset is addressed. Hopefully, our study not only provides new insights and clues in the interaction among cytokines and lipid metabolism, also paves the way to the understanding of metabolic physiology, thereby enabling progress towards prevention and treatment of the metabolic abnormalities.

## Data Availability

All data generated or analysed during this study are included in this published article and supported by our previous reports cited at relevant places within the text as refs^14–19^.
